# Chiral Phonons in 2D Halide Perovskites

**DOI:** 10.1021/acs.nanolett.5c01708

**Published:** 2025-06-16

**Authors:** Mike Pols, Geert Brocks, Sofía Calero, Shuxia Tao

**Affiliations:** † Materials Simulation & Modelling, Department of Applied Physics and Science Education, 3169 Eindhoven University of Technology, 5600 MB Eindhoven, The Netherlands; ‡ Computational Chemical Physics, Faculty of Science and Technology and MESA+ Institute for Nanotechnology, 3230 University of Twente, 7500 AE Enschede, The Netherlands

**Keywords:** metal halide perovskites, density functional theory, machine-learning force fields, chirality, phonons, chiral phonons, angular momentum

## Abstract

Phonons in chiral crystal structures can be circularly polarized, making them chiral. Chiral phonons carry angular momentum, which is observable in heat currents, and, via coupling to electron spin, in spin currents. Two-dimensional (2D) halide perovskites, versatile direct band gap semiconductors, can easily form chiral structures by incorporating chiral organic cations. As a result, they exhibit phenomena such as chirality-induced spin selectivity (CISS) and the spin Seebeck effect, although the underlying mechanisms remain unclear. Using on-the-fly machine-learning force fields trained against density functional theory calculations, we confirm the presence of chiral phonons, a potential key factor for these effects. Our analysis reveals that low-energy phonons, originating from the inorganic framework, primarily exhibit chirality. Under a temperature gradient, these chiral phonons generate substantial angular momentum, leading to experimentally observable effects. These findings position chiral 2D perovskites as a promising platform for exploring the interplay among phononic, electronic, spintronic, and thermal properties.

## Graphical abstract

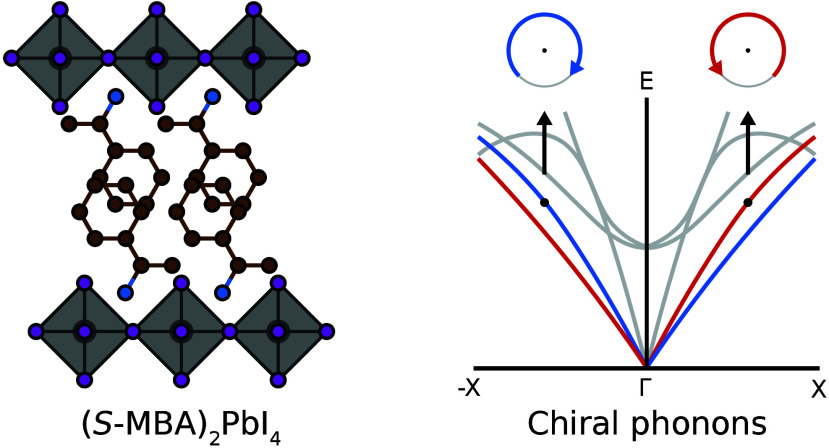

Chirality, a fundamental property of matter, manifests itself across disciplines, from biology to optical and quantum materials. Chiral molecules or crystals exist in mirror-image forms that cannot be superimposed, giving rise to distinctive properties such as optical rotation and circular dichroism (CD), observed in materials such as tellurium (Te).^1,2^ Beyond optical properties, chirality is also found to affect the properties of charge carriers, i.e. electrons and holes, in materials, for instance in chirality-induced spin selectivity (CISS),^3^ fermions in graphene,^4^ Weyl semimetals,^5^ and topological insulators.^6^ Moreover, chirality also emerges in bosons, as confirmed by the discovery and characterization of chiral phonons in two-dimensional (2D) transition metal dichalcogenides (TMDs),^7,8^ Moiré superlattices^9,10^ and three-dimensional (3D) materials like α-HgS,^11^ α-SiO_2_,^12^ and Te.^13^

Chiral phonons have a nonzero angular momentum.^7^ Under thermal equilibrium the phonon occupancies are such that their total angular momentum averages to zero. However, in heat transport experiments out-of-equilibrium distributions of chiral phonons are created, enabling them to carry an observable amount of angular momentum,^14^ analogous to the Edelstein effect in electrical transport.^15–17^ This nonzero phonon angular momentum can be experimentally measured as the crystal displays a recoil rotational motion, the Einstein-de Haas effect,^18–20^ or via the magnetic moment associated with the angular momentum.^21^ Alternatively, the phonon angular momentum can couple with electron spin,^22^ leading to the generation and detection of spin currents,^23^ as observed in the spin Seebeck effect.^24,25^

Despite significant advances, chiral effects in crystals are typically dictated by rigid crystal structures, making them difficult to manipulate. Hybrid organic–inorganic 2D halide perovskites form a class of materials with unprecedented tunability, as their properties can be adjusted by substituting different ions. By incorporating chiral organic cations, a chiral crystal structure is formed,^26,27^ due to a transfer of structural chirality to the metal halide framework.^28–30^ As a result, these materials exhibit remarkable chiroptical properties,^31^ such as optical rotation and CD,^32,33^ the emission and detection of circularly polarized light,^34–37^ and spin-polarized currents without the need for magnetism (CISS).^38,39^ Furthermore, the suggested link between chiral phonons and the spin Seebeck effect further highlights their potential as a platform for coupling optical, electronic, and thermal properties.^25^

Our work focuses on elucidating the chiral properties of phonons in 2D halide perovskites, a promising yet underexplored domain. Using machine-learning force fields (MLFFs) trained against density functional theory (DFT) calculations,^30^ we characterize the phonon modes of chiral 2D MBA_2_PbI_4_ at the harmonic level. By calculating the angular momentum of phonons as a function of their propagation direction, and mimicking heat transport experiments using the Boltzmann transport equation, we quantify the angular momentum generated under thermal gradients. Our findings demonstrate a significant anisotropy in the generated angular momentum, predominantly within the lead iodide planes, the magnitude of which can be altered by changing the crystal axis along which the gradient is applied. This anisotropic behavior paves the way for tailored spintronic and thermoelectric applications.

To assess the character of the lattice vibrations in chiral (*S*-MBA)_2_PbI_4_, we show its phonon density of states (DOS) in Figure 1. The details of these calculations can be found in Supporting Notes 1–3. Analogous to three-dimensional (3D) perovskites,^40,41^ we can identify three energy regions (Figure 1a); (i) a low-energy region, 0–25 meV, (ii) an intermediate-energy region, 25–210 meV, and (iii) a high-energy region, 375–425 meV. Considering the contributions of the different atoms in the crystal lattice, the energy regions can be associated with the motion of different parts of the crystal lattice.

**Figure 1:**
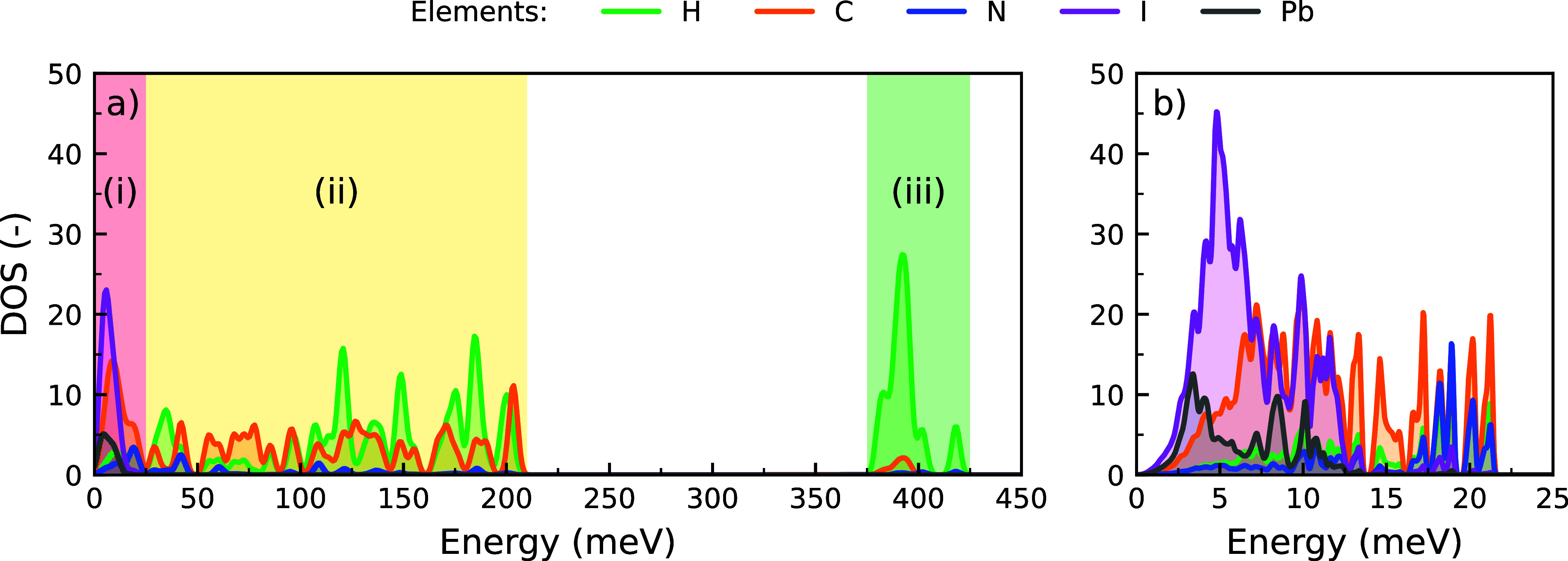
(a) Phonon density of states (DOS) of (*S*-MBA)_2_PbI_4_ with the (i) low-energy, (ii) intermediate-energy, and (iii) high-energy regions colored red, yellow, and green, respectively. (b) Close-up of the low-energy region (0–25 meV). Gaussian broadenings of 2.0 and 0.1 meV were used in the full and detailed DOS, respectively.

The low-energy region (i), shown in Figure 1b, is primarily the result of vibrations in the inorganic framework ([PbI_4_]^2–^), in particular in the lower half of that energy region. Some motion of the organic cations (MBA^+^) is mixed in, especially in the upper half of that energy region, due to coupling between the inorganic framework and the organic molecules.^42^ The corresponding vibrations involve motions of the cations as a whole. Indeed, comparing the phonon DOS of different 2D perovskites (Supporting Note 4), we find that the contribution of the organic cations at low energies scales with their size and mass.

In contrast, the two higher energy regions are the result of vibrations within the organic cations. The intermediate-energy region (ii) is associated with torsional or bending motion of molecular fragments or, for example, C–C and C–N stretch vibrations. The high-energy modes (iii) correspond to the stretch vibrations of C–H and N–H bonds in the organic cations.

In heat transport and electron–phonon coupling the low-energy phonons are particularly relevant. The phonon dispersion in the low-energy region of (*S*-MBA)_2_PbI_4_ (0–5 meV) are shown in Figure 2. We specifically focus on the dispersions in the in-plane Γ–X and Γ–Y as well as out-of-plane Γ–Z directions of the inorganic layers within the 2D perovskite (Figure 2a-d). The absence of any imaginary modes in the phonon dispersion indicates that the *P*2_1_2_1_2_1_ crystal structure of (*S*-MBA)_2_PbI_4_ observed in experiments,^28^ is a stable energy minimum.

**Figure 2:**
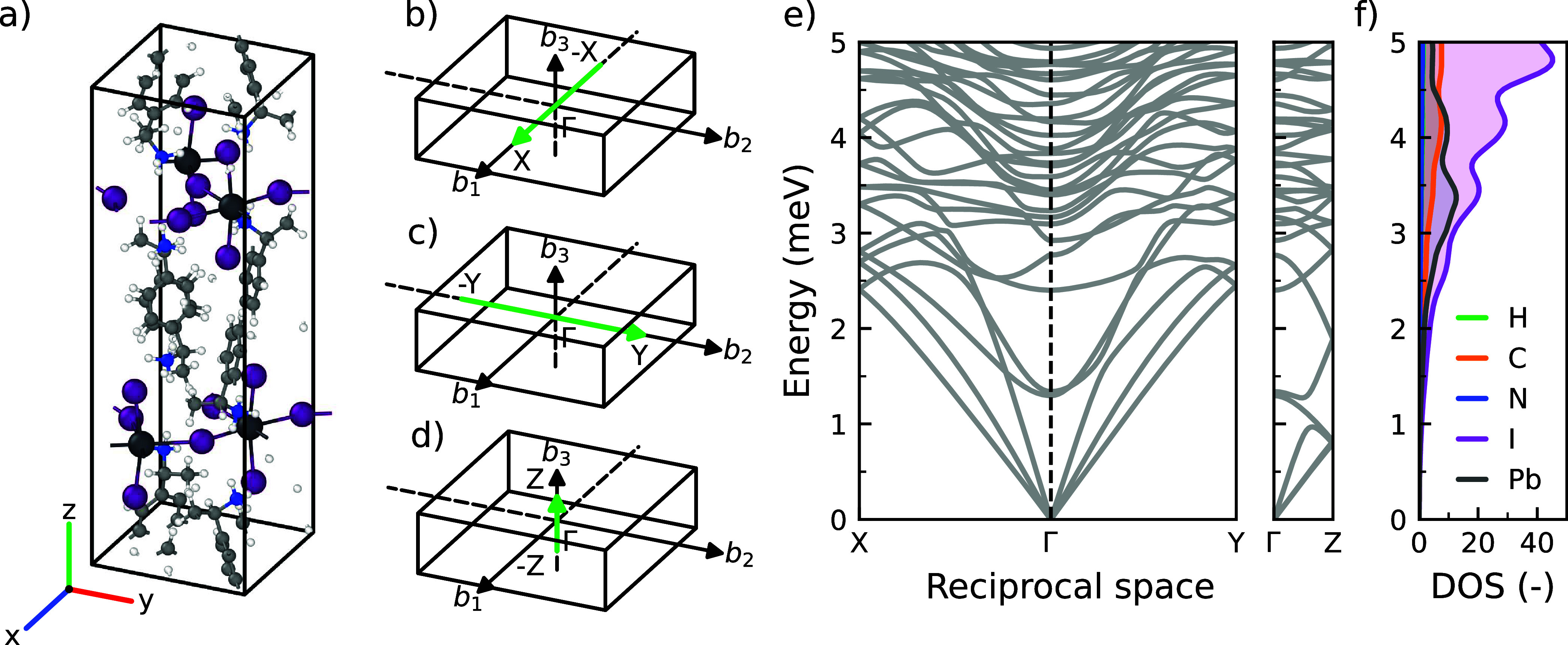
(a) Unit cell of (*S*-MBA)_2_PbI_4_ with the *P*2_1_2_1_2_1_ space group. Hydrogen (H), carbon (C), nitrogen (N), iodine (I), and lead (Pb) are represented with white, gray, blue, purple, and dark gray spheres, respectively. Brillouin zone of (*S*-MBA)_2_PbI_4_, with paths along the (b) *b*_1_-axis (Γ–X), (c) *b*_2_-axis (Γ–Y), and (d) *b*_3_-axis (Γ–Z). Special points are X = ( $\frac{1}{2}$ , 0, 0), Y = (0, $\frac{1}{2}$ , 0), and Z = (0, 0, $\frac{1}{2}$ ), with −X = (- $\frac{1}{2}$ , 0, 0). (e) Phonon dispersion and (f) density of states (DOS) of the low-energy region (0–5 meV). The DOS is broadened using a Gaussian smearing of 0.1 meV.

Focusing on the acoustic phonons (Figure 2e-f), we find that in the in-plane directions (Γ–X and Γ–Y), the three acoustic phonon branches are nondegenerate and have different group velocities. In contrast, we observe a near degeneracy between the two TA modes of the acoustic phonons in the out-of-plane direction (Γ–Z). Comparing the average group velocities of the acoustic phonons in the three directions (*v̅* ^Γ‑X^ = 1861.3 ms^–1^, *v̅* ^Γ‑Y^ = 1796.3 ms^–1^, and *v̅* ^Γ‑Z^ = 1669.2 ms^–1^), we find that they are actually quite similar, which indicates a surprisingly small anisotropy. We find a similarly low anisotropy in the phonon group velocities of BA_2_PbI_4_ and PEA_2_PbI_4_ in Supporting Note 5, which is in agreement with previous findings.^43^

A phonon eigenmode with wave vector **q** and mode index σ is described by a polarization vector **e**_i,**q**,σ_, with *i* labeling the atoms in the unit cell. The polarization vectors are normalized over all atoms of the unit cell, so that ∑_*i*=1_ **e**_i,**q**,σ_^†^ **e**_i,**q**,σ_ = 1. The circular polarization of the phonon modes can be quantified by calculation the phonon circular polarization as

1 $$s_{q,\sigma }^{\alpha }=\overset{N}{\underset{i=1}{\sum }}e_{i,q,\sigma }^{†}S^{\alpha }e_{i,q,\sigma }$$

where *S* ^α^ (α = *x*, *y*, *z*) are the spin-1 matrices on a Cartesian basis. The magnitude and sign of the circular polarization of an eigenmode determine the chirality or handedness of the phonon, with 0 < *s*_**q**,σ_^α^ ≤ +1 and – 1 ≤ *s*_**q**,σ_^α^ < 0 indicating a right- and left-handed phonon mode, respectively. Achiral phonon modes, with a linear polarization, such as longitudinal modes, have no circular polarization (*s*_**q**,σ_^α^ = 0).

The circular polarization can, in principle, be measured with respect to any arbitrary axis α. However, some components will be zero for symmetry reasons. For instance, in the crystal structure of (*S*-MBA)_2_PbI_4_, space group *P*2_1_2_1_2_1_, for phonons propagating in a direction along one of the crystal axes **q** = (*q*_*x*_, 0, 0), (0, *q*_*y*_, 0) or (0, 0, *q*_*z*_), only the corresponding *x*-, *y*-, or *z*-component of *s*_**q**,σ_^α^ is nonzero. In the current work we focus on those phonons propagating either in *x*-, *y*-, or *z*-directions. To calculate *s*_**q**,σ_^α^ we follow the procedures outlined in earlier work.^7,44^ Additional details are provided in Supporting Note 6.

The dispersion of phonons in the low-energy region of (*S*-MBA)_2_PbI_4_, propagating along the *x*-, *y*-, and *z*-axis, as well as their respective chirality, is shown in Figure 3. Phonons propagating in the positive and negative direction along the different axes are shown in separate panels. Figure 3 demonstrates that in all directions chiral phonons can be found. One observes that phonons propagating in opposite directions, for instance, Γ–X and −X−Γ, see (Figure 3a), have an opposite circular polarization, i.e. *s*_**q**,σ_^*x*^ = –*s*_–**q**,σ_^*x*^. This is a consequence of time-reversal symmetry,^14^ and is similar to the relation between spin–orbit split bands in an electronic band structure. Indeed, a similar coupling between the phonon propagation direction and its polarization has been observed in both Te and α-SiO_2_.^44^

**Figure 3:**
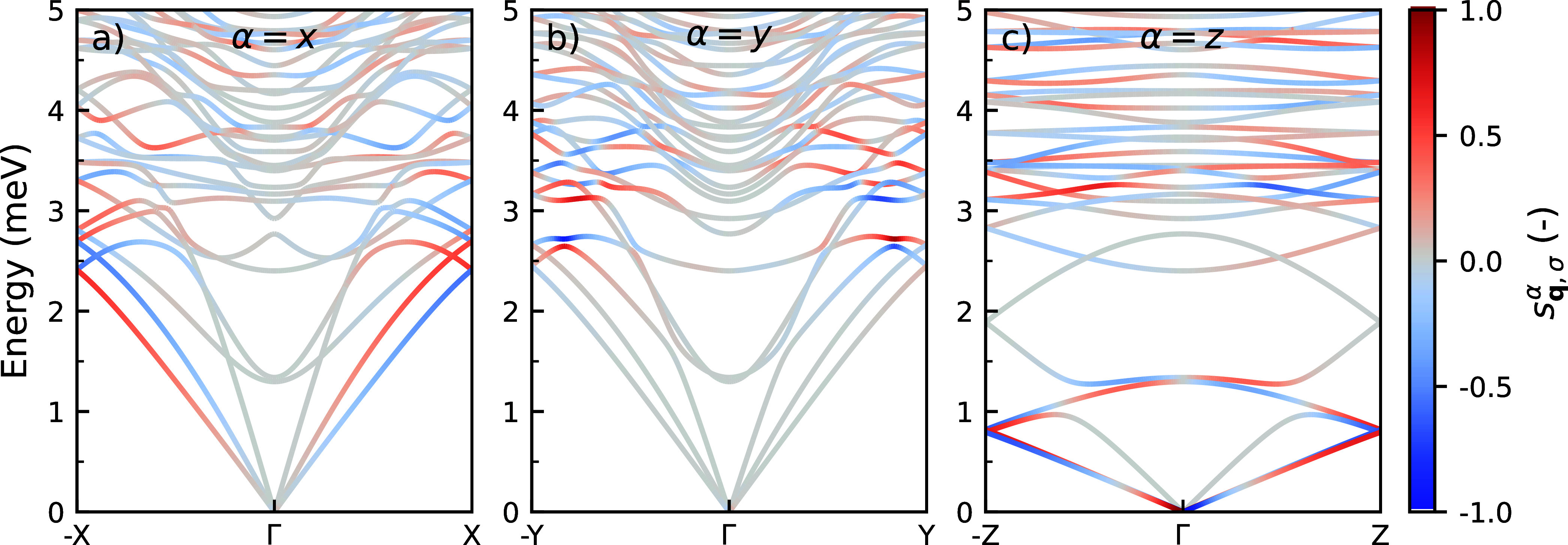
Phonon dispersion along the (a) *x*-axis (−X−Γ–X), (b) *y*-axis (−Y−Γ–Y), and (c) *z*-axis (−Z−Γ–Z) of (*S*-MBA)_2_PbI_4_. Phonon branches are color-coded with the circular polarization of the phonon modes. Red, blue, and gray are used to represent right-handed (*s*_**q**,σ_^α^ > 0), left-handed (*s*_**q**,σ_^α^ < 0), and nonpolarized (*s*_**q**,σ_^α^ = 0) phonon modes, respectively.

By examining the chirality of the phonons across the whole spectrum, we find that the chiral phonon modes are primarily found in the low-energy region of the phonon spectrum of (*S*-MBA)_2_PbI_4_. In this region (0–25 meV), phonons can possess a substantial chirality (|*s*_**q**,σ_^α^| ≥ $\frac{1}{5}$ ), whereas that of higher energy phonons (>25 meV) is negligible, as shown in Figure S8 in Supporting Note 6. Generally speaking, the phonon chirality appears to increase for phonons with wave vectors approaching the zone boundaries. In the *x*-direction (Figure 3a), the two lowest acoustic branches show appreciable chirality, as do several of the low-energy optical branches. Chirality in the *y*-direction is predominantly observed in the optical modes near the zone boundary (Figure 3b). The two lowest acoustic modes in the *z*-direction seem to show appreciable chirality (Figure 3c), but this is slightly misleading, as they are almost degenerate, and their chirality sums up to zero.

As mentioned earlier, the low-energy phonons are those phonons which heavily involve motions of the atoms within the inorganic framework. In previous work, we established that this inorganic framework has chiral structural distortions, resulting from the transfer of chirality from the organic cations to the inorganic framework.^30^ In contrast, achiral 2D perovskites were found to lack such chiral distortions and chiral phonon modes. Indeed, for achiral 2D perovskites, i.e. (*rac*-MBA)_2_PbI_4_, BA_2_PbI_4_, or PEA_2_PbI_4_, we do not observe any circular polarization of the phonon modes, as shown in Supporting Note 6.

To support the evidence of the relation between structural chirality and phonon chirality, we have also compared the phonons in the two enantiomers of MBA_2_PbI_4_ in Supporting Note 7. Whereas the phonon dispersions are identical for the two enantiomers, we observe that phonons propagating in the same direction in each enantiomer have opposite polarization; for each phonon branch, a right-handed phonon in (*S*-MBA)_2_PbI_4_ becomes a left-handed phonon in (*R*-MBA)_2_PbI_4_, and vice versa. We propose that the chiral distortions within the inorganic framework,^30^ which can readily be tuned through compositional engineering,^29,45^ play a crucial role in the emergence of circularly polarized phonons in 2D perovskites.

Figure 4 shows an example of the atomic motions of chiral phonons in (*S*-MBA)_2_PbI_4_. It illustrates the atomic motion of the lowest four phonon modes at the point O = ( $\frac{2}{5}$ , 0, 0) along the in-plane Γ–X path. Modes 1, 2, and 4 are chiral, whereas mode 3 is achiral. The chiral modes exhibit an elliptical motion in the *yz*-plane, perpendicular to their propagation direction (*x*-direction). The achiral mode exhibits a linear oscillatory motion within this plane. Comparing the two lowest phonon modes, 1 and 2, we see that the semimajor axis of the elliptical motion for these two modes has a different orientation; mode 1 has it oriented along the *z*-axis, whereas it is parallel to the *y*-axis for mode 2. A similar analysis can be found in Supporting Note 7 for the phonons propagating along the Γ–Y and Γ–Z paths.

**Figure 4:**
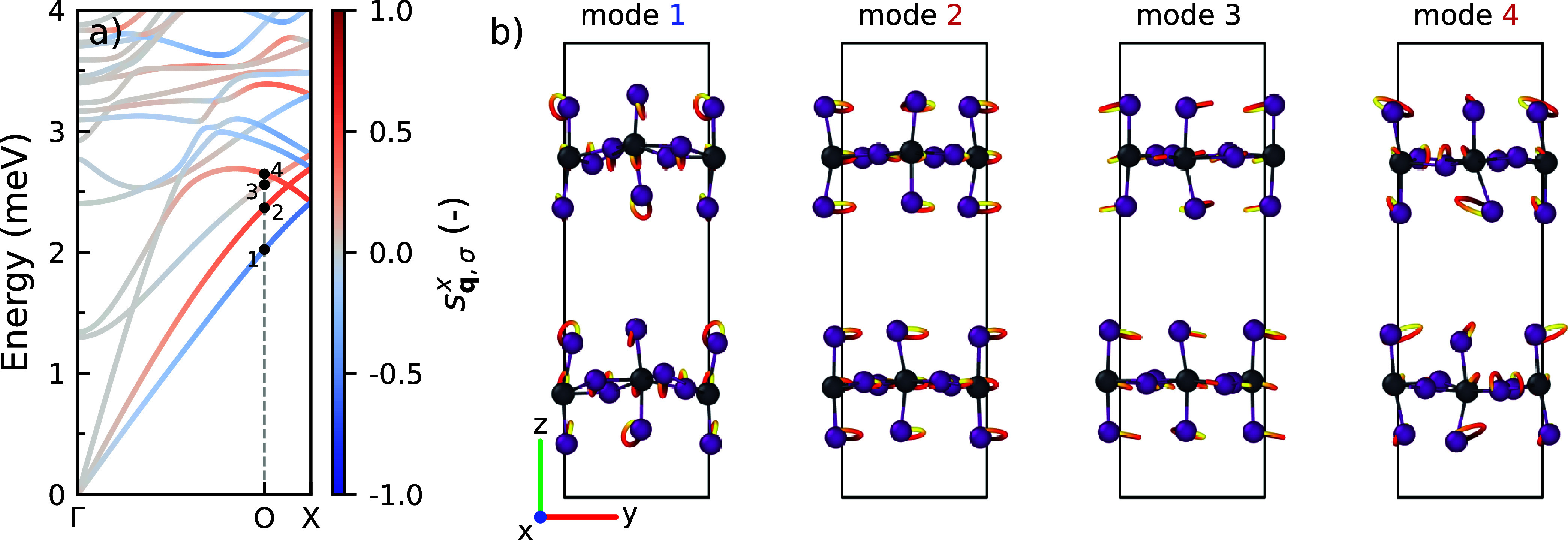
(a) Phonon dispersion and (b) atomic motion in the selected phonon modes at O = ( $\frac{2}{5}$ , 0, 0) in (*S*-MBA)_2_PbI_4_. All mode numbers are colored to indicate the circular polarization: red (right-handed), black (nonpolarized), and blue (left-handed). The atoms follow the trajectories from red to yellow as time progresses.

Having predicted the presence of chiral phonons in 2D halide perovskites, one needs a way to establish them experimentally. Figure 3 demonstrates that in every phonon branch σ phonons moving in opposite directions, i.e. ±**q**, exhibit opposite chirality, such that *s*_**q**,σ_^α^ = –*s*_**–q**,σ_^α^. In thermal equilibrium, their Bose–Einstein occupation numbers *f*_0_(ω_**q**,σ_) are equal, since for every mode ω_**q**,σ_ = ω_–**q**,σ_, which means their total chirality sums to zero.

However, the phonon distribution can be driven out-of-equilibrium by applying a temperature gradient along arbitrary directions, which generates a heat flux. This breaks the symmetry between occupations of the right- and left-moving modes, i.e. *f*(ω_**q**,σ_) ≠ *f*(ω_–**q**,σ_), and generates a phonon distribution with a nonzero net chirality. The effect is analogous to the Edelstein effect in electronic transport. As established earlier, phonon chirality is defined by a nonzero phonon circular polarization (eq 1). As the latter is a form of angular momentum, it is possible to measure it, either directly, using the Einstein-de Haas effect,^46^ or indirectly, via a coupling between phonons and magnetic moments, and the inverse spin-Hall effect.^23^

To calculate the angular momentum, we follow the procedure as formulated by Hamada et al.^14^ Heat transport is described with the Boltzmann transport equation, which is linearized under the assumption of a sufficiently small temperature gradient. In principle, each phonon mode (**q**,σ) has its own lifetime τ_**q**,σ_. However, calculating these individual lifetimes, for instance by accounting for anharmonic scattering processes, is currently computationally infeasible for a system of this size. Then it is common practice to approximate τ_**q**,σ_ with a single uniform relaxation time τ.^14^ Under these conditions, the components of the angular momentum are given by

2 $$J^{\mathrm{ph},\alpha }=-\frac{\hbar \tau }{V}\underset{q,\sigma ;\beta =x,y,z}{\sum }s_{q,\sigma }^{\alpha }v_{q,\sigma }^{\beta }\frac{\partial f_{0}\left(\omega_{q,\sigma }\right)}{\partial T}\frac{\partial T}{\partial x^{\beta }}\equiv \underset{\beta }{\sum }\alpha^{\alpha \beta }\frac{\partial T}{\partial x^{\beta }}$$

where *J* ^ph,α^ (α = *x*, *y*, *z*) are the components of the total phonon angular momentum per unit volume, ℏ is the reduced Planck constant, τ is the phonon relaxation time, *V* the unit cell volume, *s*_**q**,σ_^α^ the phonon circular polarization, *v*_**q**,σ_^β^ (β = *x*,*y*,*z*) the components of the phonon group velocity, and *f*_0_ the Bose–Einstein distribution. The response tensor of the material is then defined by α^*αβ*^. It is a second rank tensor obeying the symmetry rules of the crystal structure, which in case of space group *P*2_1_2_1_2_1_ (point group *D*_2_) gives off-diagonal elements of zero, α^αβ^ = 0 (α ≠ β), and unequal diagonal elements, α^*xx*^ ≠ α^*yy*^ ≠ α^*zz*^.^14^ A more detailed discussion on the calculation of this quantity can be found in Supporting Note 8, as well as details on the convergence of the results.

Based upon the calculated spectrum of phonon modes in (*S*-MBA)_2_PbI_4_ and their chirality, we have calculated the induced angular momentum density according to eq 2 as a function of temperature. The results are shown in Figure 5. Because of the symmetry of the structure, the induced angular momentum is parallel to the applied temperature gradient. In all directions, we observe that a gradient in the temperature results in the generation of a nonzero angular momentum. At low temperatures (<150 K), the angular momentum shows a strong dependency on temperature, but for higher temperatures (>150 K), it becomes independent of the temperature. This merely confirms that the low-energy phonons in (*S*-MBA)_2_PbI_4_ cause the chiral effect, as for *k*_B_ *T*≫ℏω_**q**,σ_, *f*_0_(ω_**q**,σ_)∝*T*, so *J* ^ph,α^ should become independent of the temperature.

**Figure 5:**
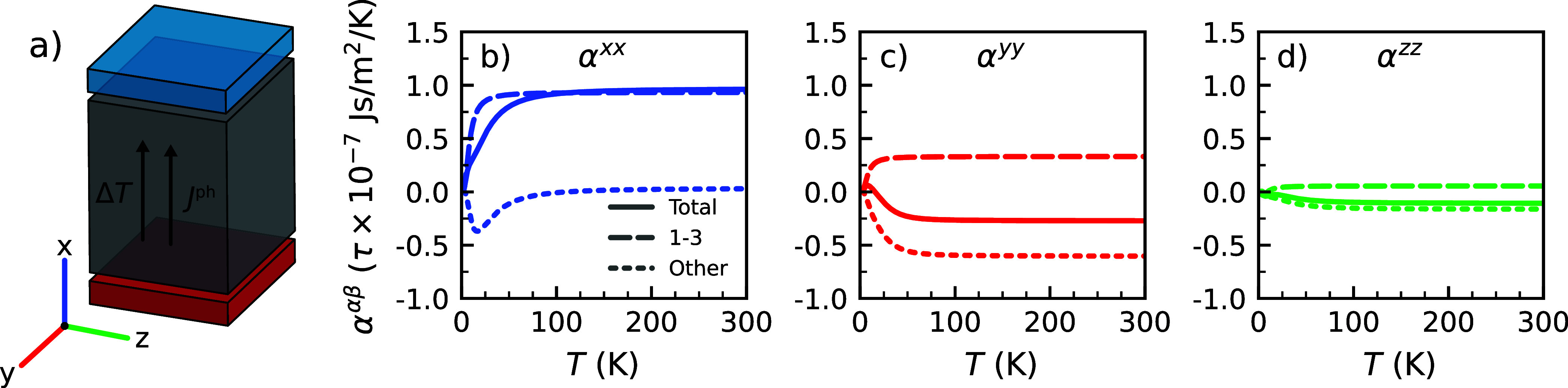
(a) Illustration of the induced angular momentum from phonons (*J* ^ph^) in (*S*-MBA)_2_PbI_4_ for a temperature gradient (Δ*T*) in the *x*-direction. Temperature dependence of the (b) α^*xx*^, (c) α^*yy*^, and (d) α^*zz*^ components of the response tensor given by solid lines. The contributions from the three lowest energy phonons and the other phonons are shown by dashed and dotted lines, respectively.

At 300 K, the induced angular momentum is largest for a temperature gradient applied in the *x*-direction (α^*xx*^ = +9.6 × τ × 10^–8^ Js m^–2^ K^–1^), with gradients applied in the *y*- and *z*-directions showing markedly lower responses and with an opposite sign (α^*yy*^ = – 2.7 × τ × 10^–8^ Js m^–2^ K^–1^ and α^*zz*^ = – 1.1 × τ × 10^–8^ Js m^–2^ K^–1^). In the current model, the angular momenta (eq 2) and the elements of the response tensor (α^αβ^) are linearly dependent on the relaxation time τ. A conservative estimate of the latter (τ ≈ 1 ps)^47^ yields an angular momentum that is large enough to be experimentally observable,^14^ particularly along the *x*-direction, either through conversion into spin signals^23^ or via torque measurements.^46^

Near the Brillouin zone (BZ) center at Γ the three lowest energy branches are formed by the acoustic modes, but near the BZ edges these hybridize with the lowest energy optical modes, so the distinction between acoustic and optical modes is blurred there, see Figure 3. Nevertheless, it is instructive to decompose the response tensors into contributions from the three lowest energy phonons and the rest. We find that above 100 K the contributions to α^*xx*^ almost completely come from the three lowest energy phonons (Figure 5b), with the higher energy optical phonons having essentially no effect. In contrast, for α^*yy*^ and α^*zz*^ (Figure 5c-d), the three lowest energy phonons and the optical phonons have similar contributions. These have opposite signs, however, which explains the smaller values of α^*yy*^ and α^*zz*^ as compared to α^*xx*^, since they cancel out.

According to eq 2, a phonon mode should have both a high chirality and high velocity to generate a substantial angular momentum. Therefore, it is not so surprising that the chiral acoustic modes provide a dominant contribution. As can be seen in Figure 3, these can be found along the Γ–X path, but not along the Γ–Y path, whereas along the Γ–Z path, modes of opposite chirality are almost degenerate, canceling their contributions.

In summary, we investigated the vibrational properties of chiral 2D perovskites, using MBA_2_PbI_4_ as a representative example. Our findings highlight that the low-energy vibrational modes (0–25 meV) are primarily associated with the inorganic framework. In contrast, the intermediate-energy (25–210 meV) and high-energy (375–425 meV) vibrations, are associated with movement of the organic cations.

A key result of our analysis is the identification of chiral phonons in the low-energy vibrational spectrum. The handedness of the chiral phonons is directly coupled to both their propagation direction and the structural chirality of the crystal. Reversing either parameter results in a corresponding reversal of the phonon handedness. This coupling enables chiral 2D perovskites to generate observable angular momentum under a temperature gradient, unveiling new functionality for these materials. Notably, our results reveal pronounced anisotropy, with angular momentum primarily generated within the lead iodide planes. This angular momentum can be modulated by adjusting the crystal axis along which the temperature gradient is applied, suggesting new possibilities for directionally controlled spintronic and thermoelectric applications.

Our work provides exciting opportunities to explore the intricate interplay between phononic, electronic, spintronic, and optical phenomena. The compositional tunability of these materials, achieved through the substitution of metal ions, halide ions, or organic cations, offers unprecedented control over their structure and the behavior of the phonons. By linking their structural chirality to vibrational, electronic, and thermal properties, these materials enable the study of complex phenomena such as chirality-induced spin selectivity (CISS), the spin Seebeck effect, and other emergent behavior. These insights deepen our understanding of fundamental chiral processes and pave the way for applications in spintronics, thermoelectrics, and quantum materials, where precise control over angular momentum and phonon polarization is crucial.

## Supplementary Material



## Notes

Computational details of density functional theory (DFT) calculations, machine-learning force field (MLFF) training, and phonon calculations; comparison of phonon density of states (DOS); phonon group velocities; circular polarization of phonons; atomic motion of phonon modes; and phonon angular momentum response tensor (PDF)
