# Supplementary material for: Chiral Phonons in 2D Halide Perovskites
Source: Nano Lett. 2025 Jun 16;25(25):10003–9. doi: 10.1021/acs.nanolett.5c01708 (PMC12203639; doi:10.1021/acs.nanolett.5c01708)
Supplement: Supplementary file 1 [file nl5c01708_si_001.pdf]

Supporting Information:
Chiral Phonons in 2D Halide Perovskites

Mike Pols,[1,*] Geert Brocks,[1,2] Sofía Calero,[1] and Shuxia Tao[1,†]

[1]*Materials Simulation & Modelling, Department of Applied Physics and Science Education, Eindhoven University of Technology, 5600 MB, Eindhoven, The Netherlands*

[2]*Computational Chemical Physics, Faculty of Science and Technology and MESA+ Institute for Nanotechnology, University of Twente, 7500 AE, Enschede, The Netherlands*

[*] m.c.w.m.pols@tue.nl

[†] s.x.tao@tue.nl

CONTENTS

1. SI Note: Density functional theory (DFT) S3

2. SI Note: Machine-learning force fields (MLFFs) S5

3. SI Note: Phonon calculations S7

4. SI Note: Density of states (DOS) S9
   A. Enantiomeric effects S9
   B. Influence of cations S9

5. SI Note: Phonon group velocities S11

6. SI Note: Chiral phonons S12
   A. Phonon circular polarization S12
   B. Circular polarization in chiral perovskites S13
   C. Circular polarization in achiral perovskites S15
   D. Circular polarization spectrum S17

7. SI Note: Phonon modes S18
   A. Atomic motion in eigenmodes S18
   B. Eigenmodes in chiral perovskites S18
   C. Eigenmodes in structural enantiomers S20

8. Phonon angular momentum S22
   A. Response tensor S22
   B. Symmetry constraints S22
   C. Convergence S23

References S24

1. SI NOTE: DENSITY FUNCTIONAL THEORY (DFT)

Density functional theory (DFT) calculations were performed using the Vienna Ab-initio Simulation Package (VASP) [1–3]. The projector-augmented wave (PAW) pseudopotentials [4] had the following valence electron configurations: H ($1s^1$), C ($2s^2 2p^2$), N ($2s^2 2p^3$), I ($5s^2 5p^5$) and Pb ($6s^2 6p^2$). In the machine-learning force fields (MLFF) the electronic exchange-correlation interactions were modeled with the meta-GGA SCAN functional [5]. For comparison, we also trained an MLFF against the Perdew, Burke and Ernzerhof (PBE) functional [6] with long range dispersion interactions accounted for by the DFT-D3(BJ) scheme [7]. In all calculations, an energy cutoff of 500 eV was used. A Γ-centered $2\times2\times1$ $\mathbf{k}$-mesh, with the single $\mathbf{k}$-point in the direction perpendicular to the inorganic layers, was used for DFT calculations of $(S\text{-MBA})_2PbI_4$, BA_2PbI_4 and PEA_2PbI_4. As such, a Γ-centered $1\times2\times2$ $\mathbf{k}$-mesh was used for $(rac\text{-MBA})_2PbI_4$.

As a starting point for the calculations, we used experimental crystal structures. An overview of the structures used is found in Table S1. All structures were optimized by allowing the atomic positions, cell shape, and cell volume to change, using an energy and force convergence criterion of 10^{-5} eV and 10^{-2} eV Å^{-1}, respectively. The optimized crystal geometries and the used $\mathbf{k}$-meshes are shown in Table S2, demonstrating good agreement between the DFT calculations and the experimental geometries.

TABLE S1. Experimental crystal structures of investigated 2D halide perovskites.

Perovskite	Space group	N_{group}	$T_{\text{exp.}}$ (K)	Database ID	References
$(S\text{-MBA})_2PbI_4$	$P2_12_12_1$	19	298	CCDC:2015617	Ref.[8]
$(rac\text{-MBA})_2PbI_4$	$P2_1/c$	14	293	CCDC:1877052	Ref.[9]
BA_2PbI_4	$Pbca$	61	100	CCDC:2018893	Ref.[10]
PEA_2PbI_4	$P\bar{1}$	2	296	CCDC:1542461	Ref.[11]

TABLE S2. Experimental, DFT-optimized, and MLFF-optimized crystal geometries for 2D halide perovskites.

Perovskite	Type	a (Å)	b (Å)	c (Å)	α (°)	β (°)	γ (°)	V (Å^3)	$\mathbf{k}$-mesh
	Exp.	8.90	9.31	28.86	90.0	90.0	90.0	2393.3	
(S-MBA)$_2$PbI$_4$	SCAN	8.87	9.19	28.76	90.0	90.0	90.0	2342.5	2×2×1
	MLFF	8.87	9.16	28.77	90.0	90.0	90.0	2337.6	
	Exp.	14.62	9.38	8.78	90.0	100.1	90.0	1185.7	
(*rac*-MBA)$_2$PbI$_4$	SCAN	14.66	9.35	8.66	90.0	100.8	90.0	1167.0	1×2×2
	MLFF	14.57	9.29	8.61	90.0	99.0	90.0	1151.5	
	Exp.	8.42	9.00	26.08	90.0	90.0	90.0	1975.6	
BA$_2$PbI$_4$	SCAN	8.45	9.00	26.10	90.0	90.0	90.0	1986.6	2×2×1
	MLFF	8.41	9.01	26.33	90.0	90.0	90.0	1994.9	
	Exp.	8.74	8.74	33.00	84.6	84.7	89.6	2498.3	
PEA$_2$PbI$_4$	SCAN	8.70	8.72	32.76	85.6	85.7	89.4	2471.5	2×2×1
	MLFF	8.71	8.74	32.59	85.4	85.3	89.4	2467.3	

2. SI NOTE: MACHINE-LEARNING FORCE FIELDS (MLFFS)

Machine-learning force fields (MLFFs) were trained against total energies, forces, and stresses from density functional theory (DFT) calculations. The training sets were automatically constructed using an on-the-fly active learning scheme together with dynamical simulations in an *NpT* ensemble [12, 13]. Local atomic environments were described using an adaptation of the smooth overlap of atomic positions (SOAP) descriptor [14], for which we employed a cutoff for the two-body radial descriptor of 6.0 Å and a cutoff of 4.0 Å for the three-body angular descriptor. The atomic positions were broadened using Gaussian distributions with a width of 0.5 Å. Both descriptors were expanded on a basis set of spherical Bessel functions and Legendre polynomials, using 8 and 6 Bessel functions for the two-body and three-body descriptors, respectively, with a maximum quantum number of angular momentum of $l_{\mathrm{max}} = 2$. The expansion coefficients of this basis set constituted the descriptor for the local atomic environments. To measure the similarity between two local atomic environments, a polynomial kernel function to power 4 was used, in which the two-body radial and three-body angular descriptor vectors were weighted by 0.1 and 0.9, respectively.

The MLFF training was initialized with a constant temperature simulation at 300 K, starting from the optimized crystal structure. This training run was followed by consecutive constant temperature simulations at 100 K and 450 K, each using the final positions and velocities of the previous run as the starting point. All constant temperature simulations were 50 ps. In the final training run, the system was cooled from 350 K to 50 K over 60 ps. The starting point for this simulation, i.e. atomic positions and velocities, was obtained using 10 ps equilibration runs with the intermediate MLFFs. During training the temperature and pressure were controlled using Parrinello-Rahman dynamics [15, 16] using friction coefficients $\gamma = 5\,\mathrm{ps}^{-1}$ and $\gamma_{\mathrm{L}} = 5\,\mathrm{ps}^{-1}$ for the atomic and lattice degrees of freedom, respectively. A time step of $\Delta t = 2$ fs and a hydrogen mass of $m_{\mathrm{H}} = 4$ u were used to enhance the sampling rate of new structures. To control the size of the force fields, the number of local reference configurations was capped at 2000, allowing configurations to be discarded once this threshold was reached. Using the training data of the final MLFFs, the models were refit onto faster descriptors without any Bayesian error estimation to speed up the evaluation for large-scale molecular dynamics production runs. Four models were trained in total. The size of the training sets for the MLFFs are shown in Supplementary Note 2.

In total we trained four machine-learning force fields (MLFFs), of which the training sets

and number of local reference configurations are summarized in Table S3. The models show good agreement with DFT calculations in describing the geometries and dynamics of the halide perovskites. Geometries optimized with the MLFFs based on the SCAN XC functional are shown in Table S2. A complete validation of the dynamics of the MLFFs is found in previous work [17]. We highlight the use of a single model for MBA^+-based perovskites, i.e. (*S*-MBA)$_2$PbI$_4$, (*R*-MBA)$_2$PbI$_4$, and (*rac*-MBA)$_2$PbI$_4$, as we found a high transferability across the various enantiomers.

TABLE S3. Size of the training set and number of local reference configurations for various machine-learning force fields (MLFFs) for 2D perovskites.

XC functional	Training structure	N_{DFT} (-)	N_{basis} (-)				
			H	C	N	I	Pb
SCAN	(*S*-MBA)$_2$PbI$_4$	972	2000	2000	563	1240	310
	PEA$_2$PbI$_4$	1030	2000	2000	507	1175	218
	BA$_2$PbI$_4$	973	2000	1983	471	1129	226
PBE+D3(BJ)	(*S*-MBA)$_2$PbI$_4$	933	2000	2000	568	1190	283

3. SI NOTE: PHONON CALCULATIONS

Harmonic phonon calculations were performed using the finite displacement method in supercells, as implemented in `phonopy` [18, 19]. The crystal structures were optimized with machine-learning force fields (MLFFs), allowing the atomic positions, cell shape, and cell volume to change until the forces are below 10^{-3} eV Å^{-1}. For all perovskites, except (rac-MBA)$_2$PbI$_4$, the interatomic force constants were computed in a 3×3×1 supercell and the phonon density of states (DOS) on a 21×21×7 Γ-centered $\mathbf{q}$-mesh. The response tensor $\alpha^{\alpha\beta}$ for (S-MBA)$_2$PbI$_4$ was calculated on the same 21×21×7 Γ-centered $\mathbf{q}$-mesh, following the procedure outlined by Hamada *et al.* [20]. For (rac-MBA)$_2$PbI$_4$, a 2×3×3 supercell and a 14×21×21 Γ-centered $\mathbf{q}$-mesh were used for the interatomic force constants and the phonon DOS, respectively.

To assess the effect of the type of exchange-correlation (XC) functional on the phonon calculations, we compared the phonon dispersion obtained using MLFFs trained against different XC functionals. In Figure S1, the phonon dispersion of (S-MBA)$_2$PbI$_4$ calculated using an MLFF trained against the SCAN and PBE+D3(BJ) XC functionals are shown. Both MLFFs give qualitatively the same phonon dispersion, with the model trained on PBE+D3(BJ) showing slightly higher energies for equivalent phonon modes. We attribute this shift towards higher energies to differences between the two XC functionals. Identically, we only observe small changes in the phonon density of states between the different functionals, as shown in Figure S2.

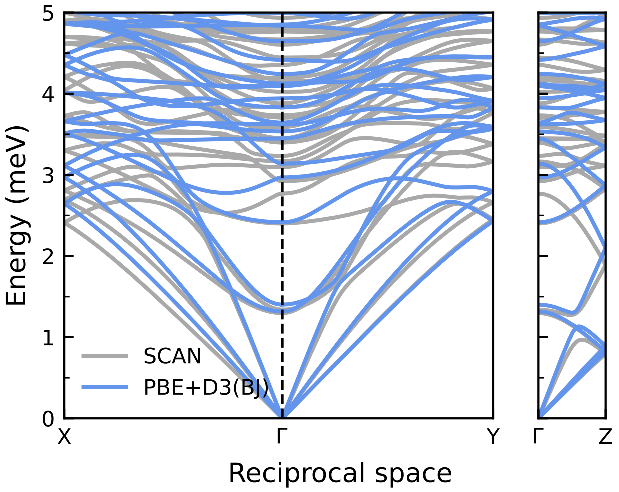

FIG. S1. Phonon dispersion of (S-MBA)$_2$PbI$_4$ computed using an MLFF trained against the SCAN and PBE+D3(BJ) XC functionals are shown in gray and blue, respectively.

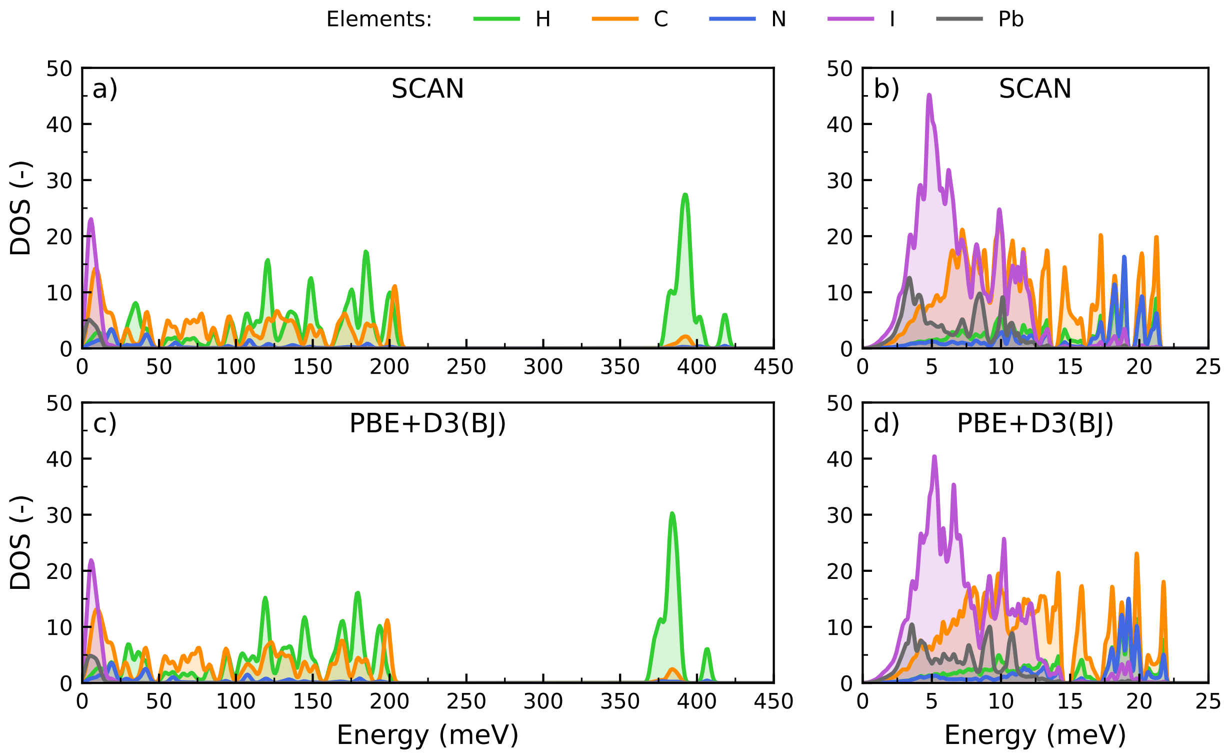

FIG. S2. Phonon density of states (DOS) of $(S\text{-MBA})_2PbI_4$ using (a-b) SCAN and (c-d) PBE+D3(BJ) exchange-correlation (XC) functional. Gaussian broadenings of 2.0 meV and 0.1 meV were used in the full and detailed DOS, respectively.

4. SI NOTE: DENSITY OF STATES (DOS)

A. Enantiomeric effects

To investigate the effect of structural enantiomers of MBA_2PbI_4 on the phonon spectrum, particularly the low enery vibrations, we compared the DOS of the enantiomers. In Figure S3 the DOS of $(S\text{-MBA})_2PbI_4$, $(R\text{-MBA})_2PbI_4$, and $(rac\text{-MBA})_2PbI_4$ are shown. The comparison shows that the DOS of the two mirror images, $(S\text{-MBA})_2PbI_4$ and $(R\text{-MBA})_2PbI_4$ (Figure S3a-b), is identical, with $(rac\text{-MBA})_2PbI_4$ (Figure S3c) only showing differences in the fine structure of the DOS. Altogether, this highlights that the structural enantiomers of a 2D halide perovskite exhibit rather similar vibrational characteristics.

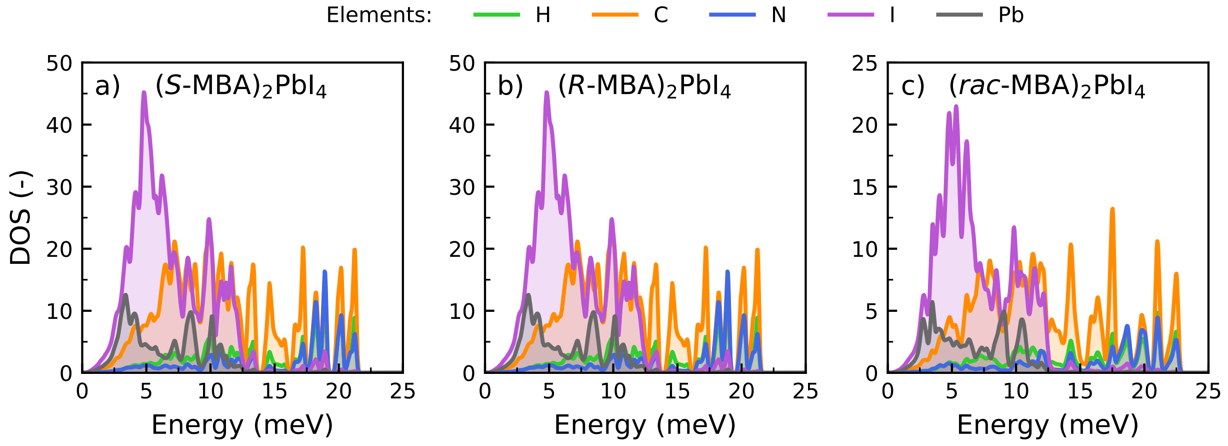

FIG. S3. Low energy (0 - 25 meV) phonon density of states (DOS) of the enantiomers of MBA^+. Phonon DOS of (a) $(S\text{-MBA})_2PbI_4$, (b) $(R\text{-MBA})_2PbI_4$, and (c) $(rac\text{-MBA})_2PbI_4$. All DOS were broadened using 0.1 meV of Gaussian broadening.

B. Influence of cations

To assess the influence of the size and mass of the organic cations on the coupling with the low energy vibrations, we compare the DOS of various 2D perovskites. In Figure S4 the DOS of $(S\text{-MBA})_2PbI_4$, PEA_2PbI_4, and BA_2PbI_4 are shown. The comparison shows that large and heavy cations (Figure S4a-b; $m_{\mathrm{MBA}^+} = 122.191\,\mathrm{u}$ and $m_{\mathrm{PEA}^+} = 122.191\,\mathrm{u}$) couple substantially more with the low energy inorganic framework vibrations than the smaller and lighter cation

(Figure S4c; $m_{\mathrm{BA}^+} = 74.147\,\mathrm{u}$).

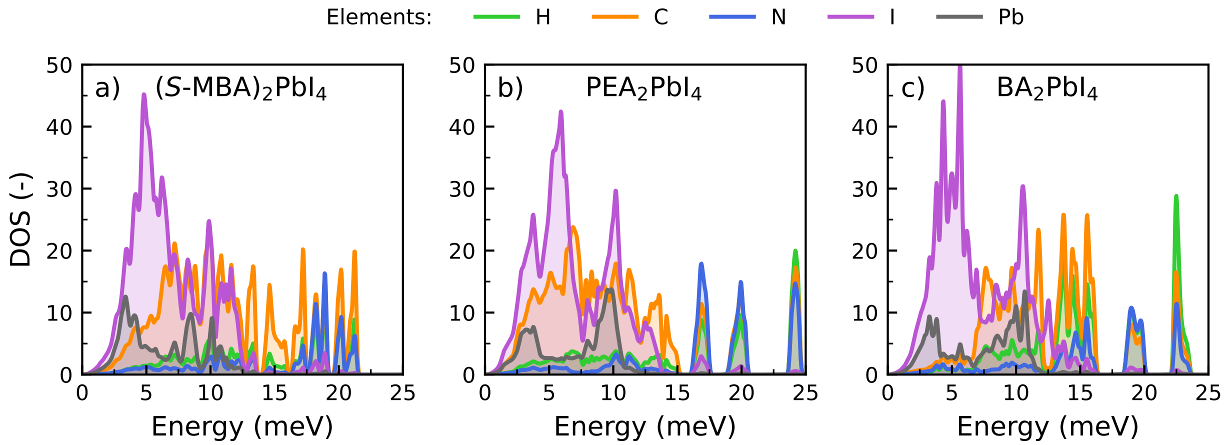

FIG. S4. Low energy (0 - 25 meV) phonon density of states (DOS) of various 2D perovskites. Phonon DOS of (a) (S-MBA)$_2$PbI$_4$, (b) PEA$_2$PbI$_4$, and (c) BA$_2$PbI$_4$. All DOS were broadened using 0.1 meV of Gaussian broadening.

5. SI NOTE: PHONON GROUP VELOCITIES

The group velocity of phonons is defined by the following relation

$$\mathbf{v}_{\mathbf{q},\sigma} = \nabla_{\mathbf{q}} \omega_{\mathbf{q},\sigma}, \tag{1}$$

which is the gradient of the angular frequency $\omega_{\mathbf{q},\sigma}$ as a function of the wave vector $\mathbf{q}$. The group velocity of the acoustic phonons is determined arbitrarily close to the Γ-point along high-symmetry paths. For the 2D halide perovskites, we determine the group velocity for the Γ−X, Γ−Y, and Γ−Z paths. The resulting group velocities of the three acoustic phonons as well as their average value ($\bar{v}$) are shown in Table S4. The investigated 2D perovskites, (S-MBA)$_2$PbI$_4$, BA$_2$PbI$_4$, and PEA$_2$PbI$_4$, all exhibit a low anisotropy in the group velocity of the phonons.

TABLE S4. Group velocities of the acoustic phonons in various 2D perovskites.

Perovskite	Mode	Group velocities		
		$v^{\Gamma-X}$ (m s^{-1})	$v^{\Gamma-Y}$ (m s^{-1})	$v^{\Gamma-Z}$ (m s^{-1})
(S-MBA)$_2$PbI$_4$	1	1151.1	1180.3	1171.2
	2	1539.3	1542.1	1175.8
	3	2893.1	2666.4	2660.6
	Average ($\bar{v}$)	1861.3	1796.3	1669.2
BA$_2$PbI$_4$	1	1221.7	1610.4	1220.8
	2	1672.9	1672.6	1612.4
	3	2719.5	2938.2	2301.7
	Average ($\bar{v}$)	1871.3	2073.7	1711.6
PEA$_2$PbI$_4$	1	1300.8	1278.7	1296.7
	2	1587.5	1587.6	1339.1
	3	2672.8	2691.4	2184.8
	Average ($\bar{v}$)	1853.7	1852.5	1606.9

6. SI NOTE: CHIRAL PHONONS

A. Phonon circular polarization

From the supercell approach, we obtain an atomic polarization vector $\mathbf{e}_{i,\mathbf{q},\sigma}$ for the ith atom in the unit cell at every wave vector $\mathbf{q}$ and mode index σ, which is of the form

$$\mathbf{e}_{i,\mathbf{q},\sigma} = (x_i, y_i, z_i)\,, \tag{2}$$

where x_i, y_i, and z_i represent the displacement of the ith atom in the x-, y-, and z-direction, respectively. Thus, every eigenmode has N such polarization vectors associated with it, one for every atom in the unit cell. The circular polarization of this eigenmode can determined using the following relation

$$s^{\alpha}_{\mathbf{q},\sigma} = \sum_{i=1}^{N} \mathbf{e}^{\dagger}_{i,\mathbf{q},\sigma} S^{\alpha} \mathbf{e}_{i,\mathbf{q},\sigma}, \tag{3}$$

where S^{α} $(\alpha = x, y, z)$ are the spin-1 matrices on a Cartesian basis.

Alternatively, this can be expressed using rotation bases, which we can define for right- and left-handed motion around an axis α. The right-handed polarization bases $|R^{\alpha}\rangle$ are

$$|R^{x}\rangle = \frac{1}{\sqrt{2}}(0, +\mathrm{i}, -1)^{\intercal} \tag{4}$$

$$|R^{y}\rangle = \frac{1}{\sqrt{2}}(+1, 0, -\mathrm{i})^{\intercal} \tag{5}$$

$$|R^{z}\rangle = \frac{1}{\sqrt{2}}(+1, +\mathrm{i}, 0)^{\intercal}. \tag{6}$$

The left-handed polarization basis $|L^{\alpha}\rangle$ can straightforwardly be obtained, by realizing this polarization basis is the complex conjugate of the right-handed polarization basis $|R^{\alpha}\rangle$ as

$$|L^{\alpha}\rangle = \left(x_j^*, y_j^*, z_j^*\right)^{\intercal}, \tag{7}$$

as such resulting in the following left-handed polarization bases around the x-, y- and z-axis

$$|L^{x}\rangle = \frac{1}{\sqrt{2}}(0, -\mathrm{i}, -1)^{\intercal} \tag{8}$$

$$|L^{y}\rangle = \frac{1}{\sqrt{2}}(+1, 0, +\mathrm{i})^{\intercal} \tag{9}$$

$$|L^{z}\rangle = \frac{1}{\sqrt{2}}(+1, -\mathrm{i}, 0)^{\intercal}. \tag{10}$$

Using these rotation bases, we can express the spin-1 matrices or circular polarization operator ($\hat{S}^\alpha$) as

$$\hat{S}^\alpha = (|R^\alpha\rangle\langle R^\alpha| - |L^\alpha\rangle\langle L^\alpha|) \,. \tag{11}$$

By putting the polarization vector in the bra-ket notation, i.e. $|e_{i,\mathbf{q},\sigma}\rangle = (x_i, y_i, z_i)^\intercal$, we can compute the circular polarization of a phonon eigenmode as

$$s^\alpha_{\mathbf{q},\sigma} = \sum_{i=1}^{N} \langle e_{i,\mathbf{q},\sigma}|\hat{S}^\alpha|e_{i,\mathbf{q},\sigma}\rangle \tag{12}$$

B. Circular polarization in chiral perovskites

In the main text, we only show the chirality of the phonon modes around their propagation direction (Figure 3); in other words, we only investigate if the phonon modes exhibited circular motion in the plane perpendicular to their propagation direction. Here we analyze the chirality of phonon modes of (S-MBA)$_2$PbI$_4$ in all directions. For example, for phonons propagating in the x-direction (Γ−X path) we assess their polarization around the x-, y-, and z-axis in Figure S5. For these high-symmetry paths, we observe that the phonons only possess a net circular polarization, and thus chirality, around their propagation direction, not in the directions perpendicular to their propagation direction. Thus, the phonons in the Γ−X, Γ−Y, and Γ−Z paths are solely polarized around the x-, y-, and z-axis, respectively.

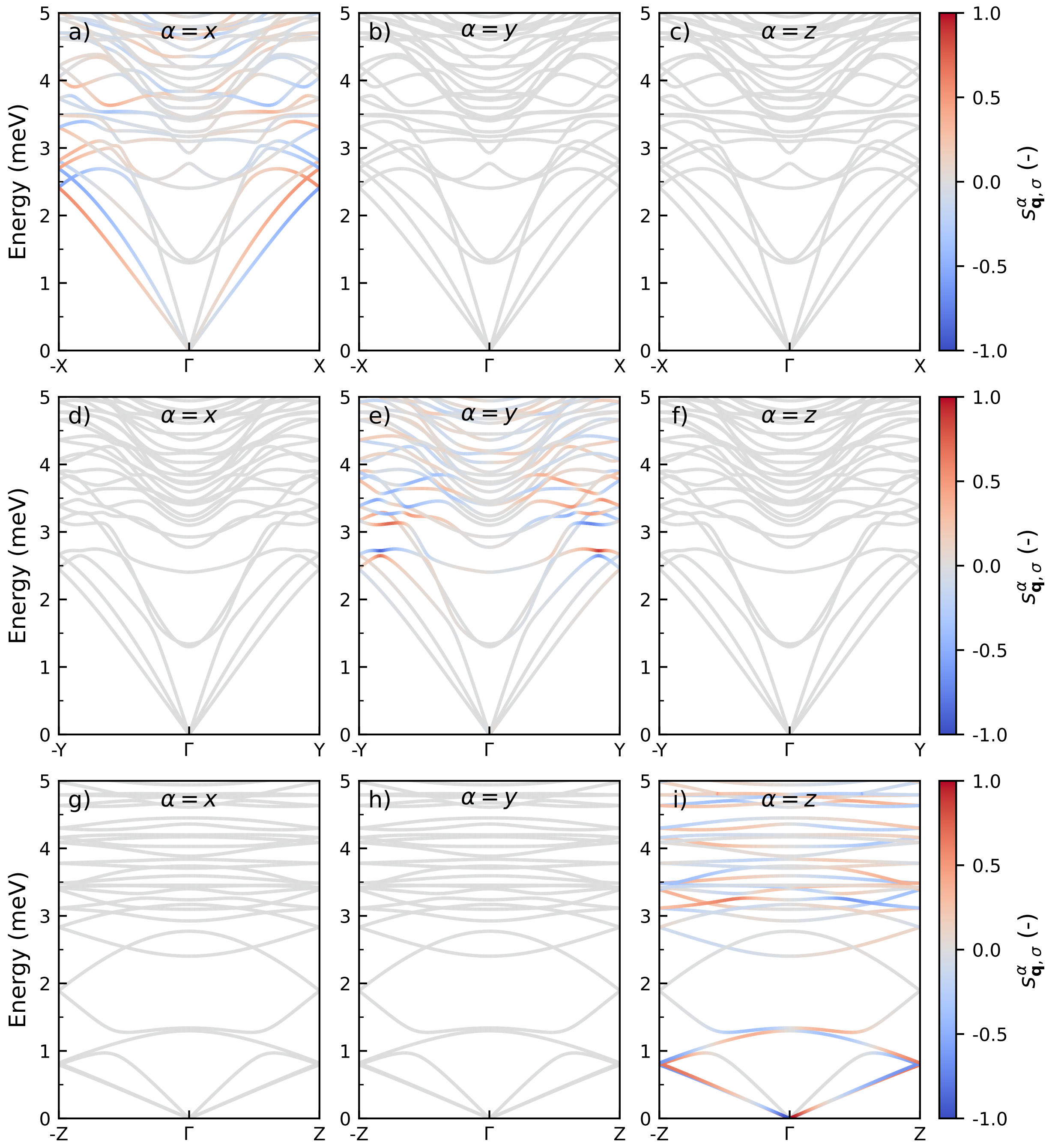

FIG. S5. Circularly polarized phonon dispersion of (S-MBA)$_2$PbI$_4$. Dispersion of phonons propagating along the (a-c) x-axis (Γ−X), (d-f) y-axis (Γ−Y), and (g-i) z-axis (Γ−Z). Phonon branches are color-coded with the circular polarization of the phonon modes, which is determined around the axis $\alpha = x$, y, or z. Red, blue, and gray are used to represent right-handed ($s^{\alpha}_{\mathbf{q},\sigma} > 0$), left-handed ($s^{\alpha}_{\mathbf{q},\sigma} < 0$), and non-polarized ($s^{\alpha}_{\mathbf{q},\sigma} = 0$) phonon modes.

C. Circular polarization in achiral perovskites

Next, we assess the circular polarization of the phonon modes in achiral perovskites. We show the circularly polarized phonon dispersion for BA_2PbI_4 and PEA_2PbI_4 in Figure S6 and for (*rac*-MBA)$_2$PbI$_4$ in Figure S7. To do so, we adopted the same naming convention for the special points as in the main text with the structures from Table S2; X = ($\frac{1}{2}$, 0, 0), Y = (0, $\frac{1}{2}$, 0), and Z = (0, 0, $\frac{1}{2}$), with -X = (-$\frac{1}{2}$, 0, 0). The phonon dispersions show that all phonon modes are achiral in the achiral perovskites.

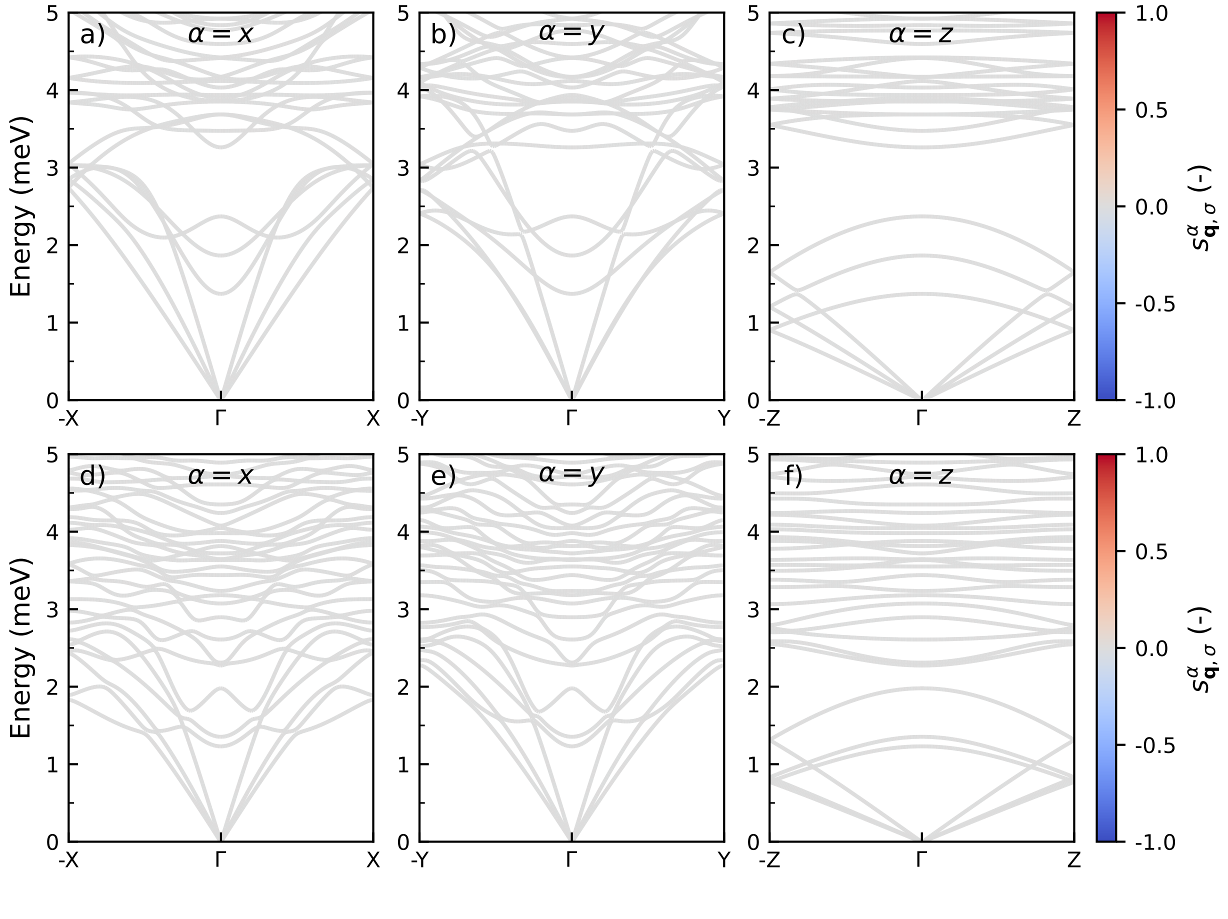

FIG. S6. Circularly polarized phonon dispersion of (a-c) BA_2PbI_4 and (d-f) PEA_2PbI_4. Phonon branches are color-coded with the circular polarization of the phonon modes, which is determined around the axis $\alpha = x$, y, or z. Red, blue, and gray are used to represent right-handed ($s^{\alpha}_{\mathbf{q},\sigma} > 0$), left-handed ($s^{\alpha}_{\mathbf{q},\sigma} < 0$), and non-polarized ($s^{\alpha}_{\mathbf{q},\sigma} = 0$) phonon modes.

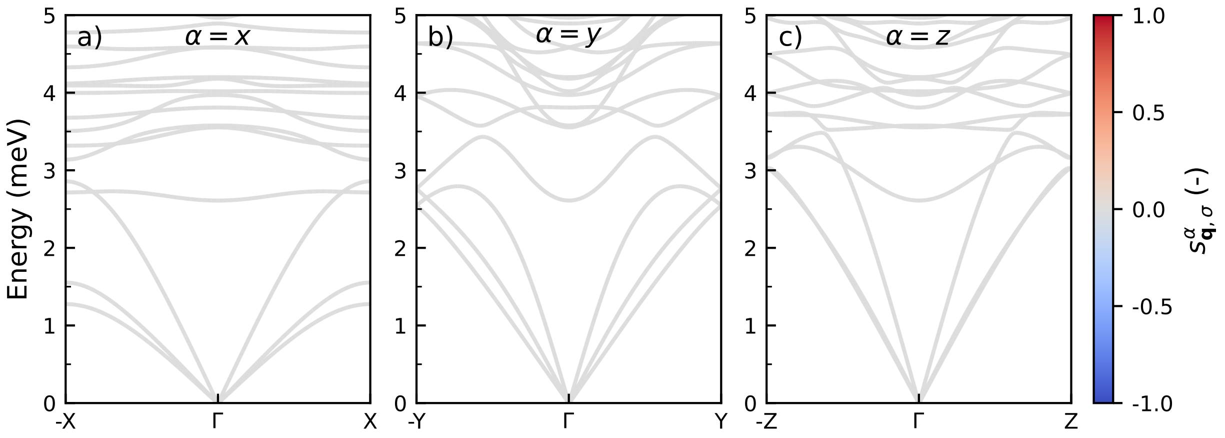

FIG. S7. Circularly polarized phonon dispersion of (a-c) $(rac\text{-MBA})_2PbI_4$. Phonon branches are color-coded with the circular polarization of the phonon modes, which is determined around the axis $\alpha = x$, y, or z. Red, blue, and gray are used to represent right-handed ($s^{\alpha}_{\mathbf{q},\sigma} > 0$), left-handed ($s^{\alpha}_{\mathbf{q},\sigma} < 0$), and non-polarized ($s^{\alpha}_{\mathbf{q},\sigma} = 0$) phonon modes.

D. Circular polarization spectrum

To clarify what part of the phonon spectrum is chiral, we plot the chirality of the phonon modes as a function of the phonon frequency in Figure S8. From this circular polarization spectrum we find that the highly chiral modes, which we characterize with ($|s^{\alpha}_{\mathbf{q},\sigma}|$ >0.2) are all low energy phonons with energies below 10 meV.

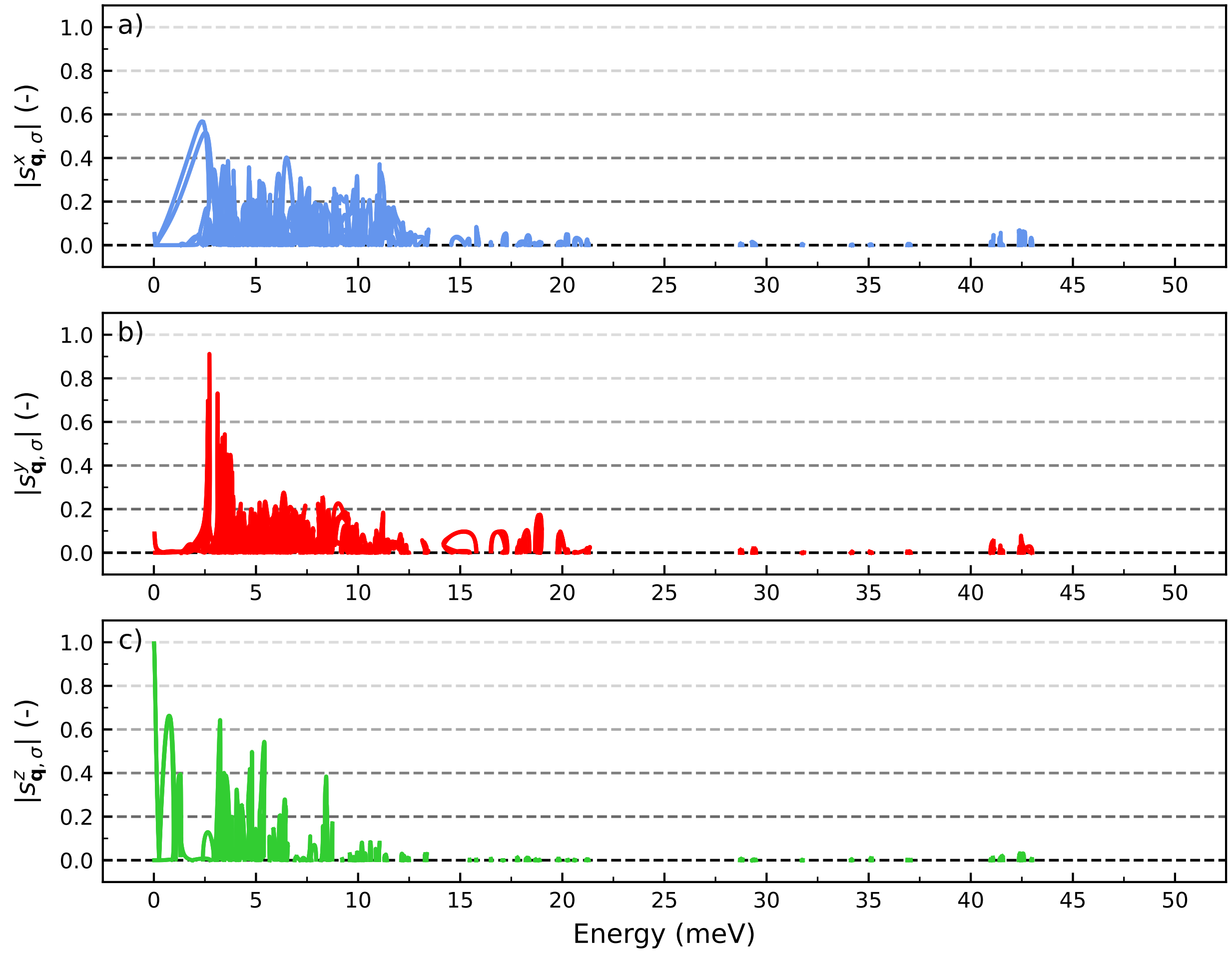

FIG. S8. Circular polarization spectrum of phonons in (S-MBA)$_2$PbI$_4$. The absolution polarization ($|s^{\alpha}_{\mathbf{q},\sigma}|$) spectrum of the phonons along the (a) Γ−X, (b) Γ−Y, and (c) Γ−Z paths. Dashed lines act as guides to the eye.

7. SI NOTE: PHONON MODES

A. Atomic motion in eigenmodes

As mentioned before, phonon eigenmodes are described at every wave vector $\mathbf{q}$ and mode index σ by a polarization vector $\mathbf{e}_{i,\mathbf{q},\sigma}$ for which i labels the atoms in the unit cell. This polarization vector has the form

$$\mathbf{e}_{i,\mathbf{q},\sigma} = (x_i, y_i, z_i)\,, \tag{13}$$

where x_i, y_i, and z_i denote the displacement of the ith atom in the x-, y-, and z-direction, respectively. Noting that the polarization vectors are written in mass normalized coordinates, they can be written in terms of real-space displacements as

$$\tilde{\mathbf{e}}_{i,\mathbf{q},\sigma} = \frac{1}{\sqrt{m_i}}\,(x_i, y_i, z_i)\,, \tag{14}$$

where m_i is the mass of the ith atom in the unit cell. The real-space displacements of the unit cell can consequently be used to determine the atomic displacements for a phonon eigenmode as

$$\mathbf{u}_{i,\mathbf{q},\sigma}(t) = \tilde{\mathbf{e}}_{i,\mathbf{q},\sigma} \mathrm{e}^{\mathrm{i}\left[\mathbf{q}\cdot\mathbf{r}_i^0 - \omega_{\mathbf{q},\sigma} t\right]}, \tag{15}$$

where t is the time, $\mathbf{r}_i^0$ is the equilibrium position of the ith atom, and $\omega_{\mathbf{q},\sigma}$ is the angular frequency of the phonon eigenmode. The time-dependent atomic positions follow from this as

$$\mathbf{r}_{i,\mathbf{q},\sigma}(t) = \mathbf{r}_i^0 + \mathbf{u}_{i,\mathbf{q},\sigma}(t)\,. \tag{16}$$

B. Eigenmodes in chiral perovskites

Having explored the atomic motion for phonons of (S-MBA)$_2$PbI$_4$ propagating along the x-axis in Figure 4, we here illustrate the atomic motion of phonons propagating along the y-axis (Figure S9) and z-axis (Figure S10). Analogous to the main text, we pick a point along the path and visualize the lowest energy modes, for the Γ−Y path P = (0, $\frac{2}{5}$, 0) and the Γ−Z path Q = (0, 0, $\frac{2}{5}$). In all chiral phonons the atoms exhibit a clear rotational motion, whereas for the achiral phonons the atoms show a linear oscillatory motion.

Analyzing the phonons propagating along the z-axis (Γ−Z), we first assess the changes in the modes upon going to phonon modes with higher energies at the Q (Figure S10a). The atomic motion associated with the six lowest energy modes is shown in Figure S10b. The lowest four

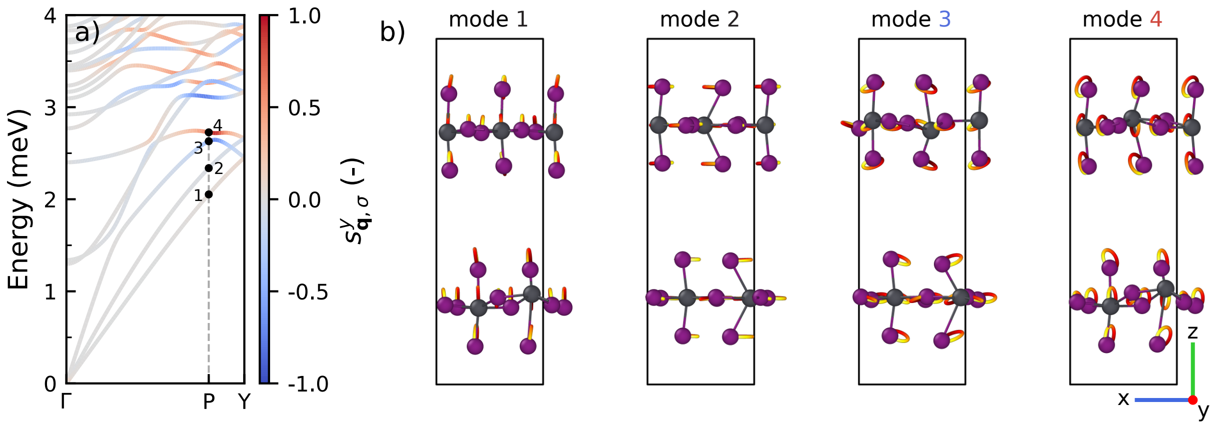

FIG. S9. Eigenmodes of in-plane phonons propagating along the y-axis (Γ−Y) in (S-MBA)$_2$PbI$_4$. (a) Phonon dispersion and (b) atomic motion in the selected phonon modes at P = (0, $\frac{2}{5}$, 0). All mode numbers are colored to indicate the circular polarization, with red (right-handed), black (non-polarized), and blue (left-handed). The atoms follow the trajectories from red to yellow as time progresses.

phonon modes at Q are highly chiral, with all modes exhibiting an elliptical planar movement in the perpendicular plane (xy-plane). The two lowest modes are of opposite chirality and nearly degenerate along the full Γ−X path. We note that the semi-major axis of the elliptical motion of the two modes is along a different direction, i.e. x-axis for mode 1 and y-axis for mode 2. The following two modes, mode 3 and mode 4, exhibit similar characteristics. The next two modes, mode 5 and 6, exhibit a linear oscillatory motion primarily in the propagation direction and are thus achiral.

We then investigate the effects of moving from point Q towards Γ along the lowest energy phonon branch. Along this branch we observe a change in the sign of the circular polarization of the phonon mode (Figure S10c), as a result of atomic motion in the phonon modes (Figure S10d). Close to Q the modes on this branch are highly chiral (mode i). Upon moving to Γ, we observe the phonon modes lose some of their right-handed circular polarization, as a result of more elliptical atomic motion (mode ii). The circular polarization completely vanishes when moving even further away from Q, where the atomic motion of the mode becomes linear (mode iii). Moving past the point of vanishing chirality, we observe the phonon mode attains an opposite chirality (mode iv).

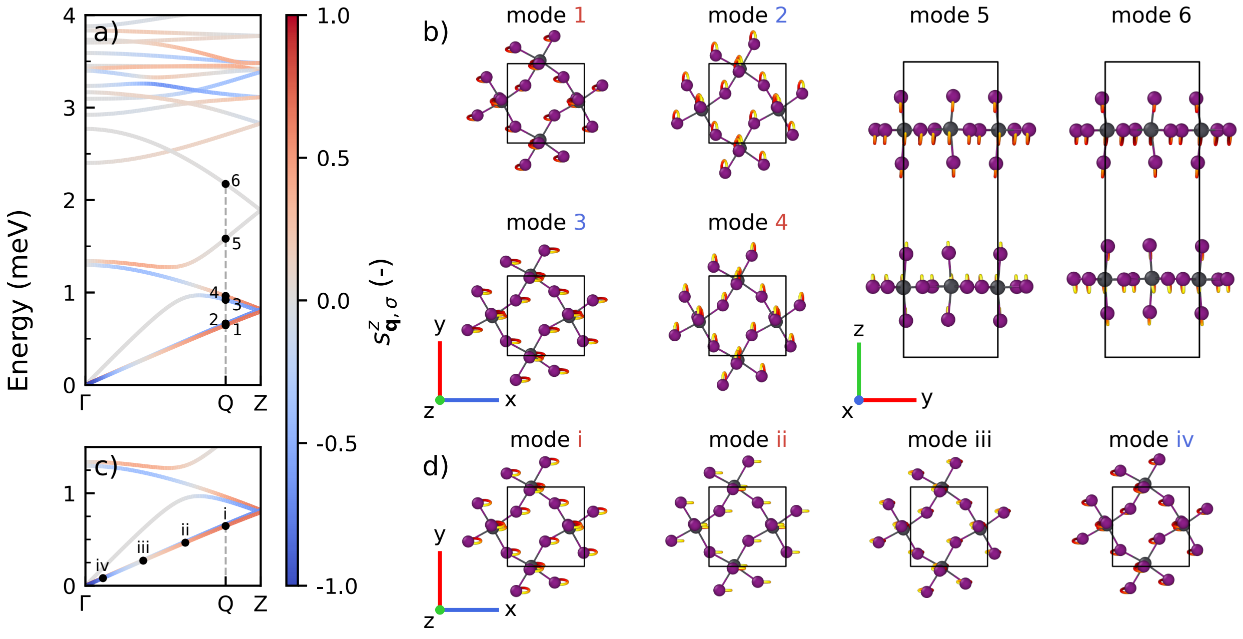

FIG. S10. Eigenmodes of out-of-plane phonons (Γ−Z) in (S-MBA)$_2$PbI$_4$. (a) Phonon dispersion and (b) atomic motion in the selected phonon modes at Q = (0, 0, $\frac{2}{5}$). (c) Phonon dispersion and (d) atomic motion in selected phonon modes along the Q−Γ path. All mode numbers are colored to indicate the circular polarization, with red (right-handed), black (non-polarized), and blue (left-handed). The atoms follow the trajectories from red to yellow as time progresses.

C. Eigenmodes in structural enantiomers

Next, to investigate the effects of structural chirality on the chirality of phonons more deeply, we compare the phonon dispersion of the two enantiomers of MBA$_2$PbI$_4$. The phonon dispersion of (S-MBA)$_2$PbI$_4$ and (R-MBA)$_2$PbI$_4$ are shown in Figure S11. We observe that phonons propagating in the same direction in each enantiomer have opposite polarization (Figure S11a-b); for each phonon branch, a right-handed phonon in (S-MBA)$_2$PbI$_4$ becomes a left-handed phonon in (R-MBA)$_2$PbI$_4$ and vice versa. As an illustration, we show the motion of the inorganic layers in the lowest energy mode at Q = (0, 0, $\frac{2}{5}$) in Figure S11c-d. Whereas the inorganic layers exhibit a counter-clockwise motion around the z-axis in (S-MBA)$_2$PbI$_4$ (Figure S11c), a clockwise motion around the z-axis is observed for the equivalent mode in (R-MBA)$_2$PbI$_4$ (Figure S11d). Interestingly, the opposite chirality of the phonon modes can be related to the structural distortions in the chiral perovskites, which between the two enantiomers has an opposite handedness, which was also observed in bulk Te [21]. Altogether this highlights the interplay between structural

chirality and the phonon handedness in such chiral materials.

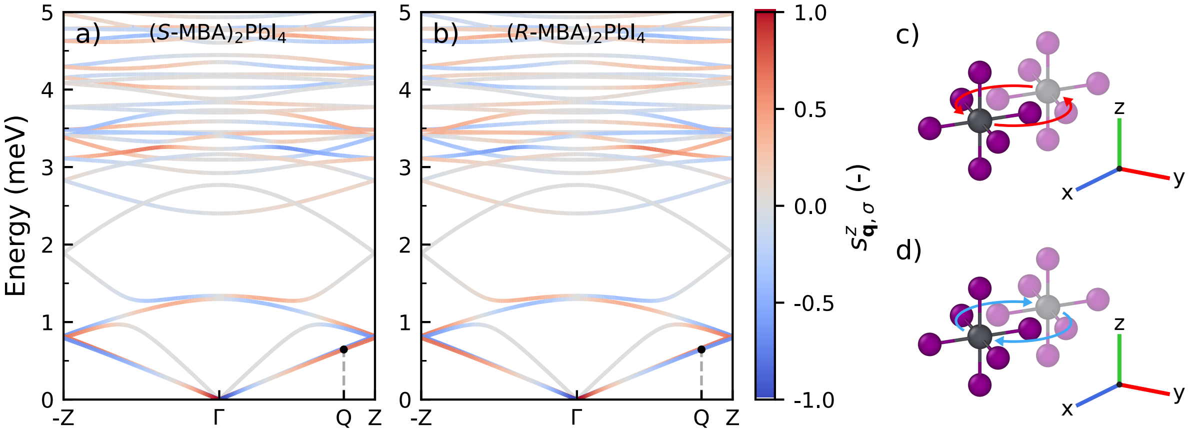

FIG. S11. Circularly polarized phonon dispersion and modes of MBA_2PbI_4. Phonon dispersion of out-of-plane ($\Gamma-$Z) phonons in (a) $(S\text{-MBA})_2PbI_4$ and (b) $(R\text{-MBA})_2PbI_4$. In the lowest energy mode at $Q = (0, 0, \frac{2}{5})$ the inorganic framework moves in a (c) counter-clockwise motion in $(S\text{-MBA})_2PbI_4$ and (d) clockwise motion in $(R\text{-MBA})_2PbI_4$ around the z-axis. Red, blue, and gray are used to represent right-handed ($s^\alpha_{\mathbf{q},\sigma} > 0$), left-handed ($s^\alpha_{\mathbf{q},\sigma} < 0$), and non-polarized ($s^\alpha_{\mathbf{q},\sigma} = 0$) phonon modes.

8. PHONON ANGULAR MOMENTUM

A. Response tensor

In equilibrium, $\mathbf{J}^{\text{ph}}$, the angular momentum per unit volume of a phonon system [22] is expressed as

$$\mathbf{J}^{\text{ph}} = \frac{\hbar}{V} \sum_{\mathbf{q},\sigma} \mathbf{s}_{\mathbf{q},\sigma} \left[f_0 \left(\omega_{\mathbf{q},\sigma} \right) + \frac{1}{2} \right], \tag{17}$$

where $\hbar$ is the reduced Planck constant, V is the unit cell volume, $f_0 = 1/\left(\exp\left[\hbar\omega_{\mathbf{q},\sigma}/k_{\text{B}}T\right] - 1\right)$ is the Bose-Einstein distribution, with k_{B} the Boltzmann constant and T the temperature. The circular polarization of a phonon is provided by the vector $\mathbf{s}_{\mathbf{q},\sigma} = (s^x_{\mathbf{q},\sigma}, s^y_{\mathbf{q},\sigma}, s^z_{\mathbf{q},\sigma})$. As a result of time-reversal symmetry, the phonon angular momentum is an odd function in $\mathbf{q}$, i.e. $\mathbf{s}_{\mathbf{q},\sigma} = -\mathbf{s}_{-\mathbf{q},\sigma}$, resulting in zero sum in Equation 17. As proposed by Hamada *et al.* [20], the system can be brought out-of-equilibrium, through the application of a temperature gradient. Following Boltzmann transport theory, the distribution function deviates from the Bose-Einstein distribution as

$$f_{\mathbf{q},\sigma} = f_0 \left(\omega_{\mathbf{q},\sigma} \right) - \sum_{\mathbf{q},\sigma;\beta=x,y,z} \tau v^{\beta}_{\mathbf{q},\sigma} \frac{\partial f_0 \left(\omega_{\mathbf{q},\sigma} \right)}{\partial T} \frac{\partial T}{\partial x^{\beta}} \tag{18}$$

where τ is the phonon relaxation time, $v^{\beta}_{\mathbf{q},\sigma}$ and $\frac{\partial T}{\partial x^{\beta}}$ are the β component of the group velocity and temperature gradient in real space. The above approximation holds for small deviations from equilibrium, under assumption that the system relaxes back to equilibrium through phonon-phonon interactions. We employ the constant relaxation time approximation for this process, which assumes τ is independent of both $\mathbf{q}$ and σ. Combining Equation 17 with Equation 18, the total angular momentum per unit volume [20, 23], generated from a temperature gradient, is

$$J^{\text{ph},\alpha} = -\frac{\hbar\tau}{V} \sum_{\mathbf{q},\sigma;\beta=x,y,z} s^{\alpha}_{\mathbf{q},\sigma} v^{\beta}_{\mathbf{q},\sigma} \frac{\partial f_0 \left(\omega_{\mathbf{q},\sigma} \right)}{\partial T} \frac{\partial T}{\partial x^{\beta}} \equiv \sum_{\beta} \alpha^{\alpha\beta} \frac{\partial T}{\partial x^{\beta}}, \tag{19}$$

with $s^{\alpha}_{\mathbf{q},\sigma}$ the α component of the phonon angular momentum and $\alpha^{\alpha\beta}$ the phonon angular momentum response tensor, with the components: $\alpha, \beta = x, y, z$.

B. Symmetry constraints

Hamada *et al.* [20] did a symmetry analysis of $\alpha^{\alpha\beta}$, the phonon angular momentum response tensor. Through the $\mathbf{q} = (0,\ 0,\ 0)$ nature of the effect, they found that the nonzero elements

of the tensor were determined by the point group symmetry of the structure, and not the space group symmetry. In case of (S-MBA)$_2$PbI$_4$, which has the space group $P2_12_12_1$ (point group $D2$), the response tensor takes the following form

$$\alpha^{\alpha\beta} = \begin{pmatrix} \alpha^{xx} & 0 & 0 \\ 0 & \alpha^{yy} & 0 \\ 0 & 0 & \alpha^{zz} \end{pmatrix}, \tag{20}$$

where all diagonal elements have unique values; $\alpha^{xx} \neq \alpha^{yy} \neq \alpha^{zz}$.

C. Convergence

To assess if the response tensor for angular momentum generation in the presence of a temperature gradient ($\alpha^{\alpha\beta}$) has converged, we compute the response tensor at 300 K for a range of reciprocal meshes. We vary these $\mathbf{q}$-meshes from 3×3×1 (N_q = 9) to 30×30×1 (N_q = 9000). In Figure S12 we show the values of α^{xx}, α^{yy}, and α^{zz} as a function of N_q, the number of points on the $\mathbf{q}$-mesh. The response tensor converges for increasingly larger reciprocal meshes, with minimal changes occurring for grids denser than 21×21×7 (N_q = 3087). Hence, the response tensors and their temperature dependence, were all computed on a 21×21×7 $\mathbf{q}$-mesh in this work.

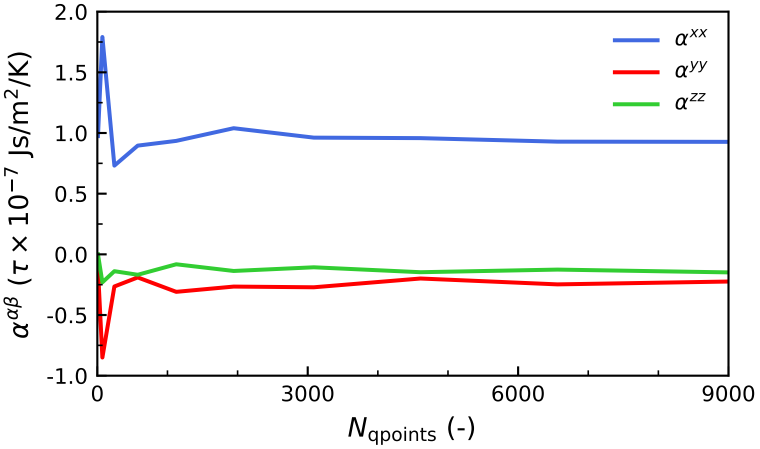

FIG. S12. Convergence of the response tensors for angular momentum generation as a result of a temperature gradient at 300 K.

[1] G. Kresse and J. Hafner, Ab Initio Molecular-Dynamics Simulation of the Liquid-Metal–Amorphous-Semiconductor Transition in Germanium, Phys. Rev. B **49**, 14251 (1994).

[2] G. Kresse and J. Furthmüller, Efficiency of Ab-Initio Total Energy Calculations for Metals and Semiconductors Using a Plane-Wave Basis Set, Comput. Mater. Sci. **6**, 15 (1996).

[3] G. Kresse and J. Furthmüller, Efficient Iterative Schemes for Ab Initio Total-Energy Calculations Using a Plane-Wave Basis Set, Phys. Rev. B **54**, 11169 (1996).

[4] G. Kresse and D. Joubert, From Ultrasoft Pseudopotentials to the Projector Augmented-Wave Method, Phys. Rev. B **59**, 1758 (1999).

[5] J. Sun, A. Ruzsinszky, and J. P. Perdew, Strongly Constrained and Appropriately Normed Semilocal Density Functional, Phys. Rev. Lett. **115**, 036402 (2015).

[6] J. P. Perdew, K. Burke, and M. Ernzerhof, Generalized Gradient Approximation Made Simple, Phys. Rev. Lett. **77**, 3865 (1996).

[7] S. Grimme, S. Ehrlich, and L. Goerigk, Effect of the Damping Function in Dispersion Corrected Density Functional Theory, J. Comput. Chem. **32**, 1456 (2011).

[8] M. K. Jana, R. Song, H. Liu, D. R. Khanal, S. M. Janke, R. Zhao, C. Liu, Z. Valy Vardeny, V. Blum, and D. B. Mitzi, Organic-to-Inorganic Structural Chirality Transfer in a 2D Hybrid Perovskite and Impact on Rashba-Dresselhaus Spin-Orbit Coupling, Nat. Commun. **11**, 4699 (2020).

[9] Y. Dang, X. Liu, Y. Sun, J. Song, W. Hu, and X. Tao, Bulk Chiral Halide Perovskite Single Crystals for Active Circular Dichroism and Circularly Polarized Luminescence, J. Phys. Chem. Lett. **11**, 1689 (2020).

[10] M. Menahem, Z. Dai, S. Aharon, R. Sharma, M. Asher, Y. Diskin-Posner, R. Korobko, A. M. Rappe, and O. Yaffe, Strongly Anharmonic Octahedral Tilting in Two-Dimensional Hybrid Halide Perovskites, ACS Nano **15**, 10153 (2021).

[11] K.-z. Du, Q. Tu, X. Zhang, Q. Han, J. Liu, S. Zauscher, and D. B. Mitzi, Two-Dimensional Lead(II) Halide-Based Hybrid Perovskites Templated by Acene Alkylamines: Crystal Structures, Optical Properties, and Piezoelectricity, Inorg. Chem. **56**, 9291 (2017).

[12] R. Jinnouchi, F. Karsai, and G. Kresse, On-the-Fly Machine Learning Force Field Generation: Application to Melting Points, Phys. Rev. B **100**, 014105 (2019).

[13] R. Jinnouchi, J. Lahnsteiner, F. Karsai, G. Kresse, and M. Bokdam, Phase Transitions of Hybrid Perovskites Simulated by Machine-Learning Force Fields Trained on the Fly with Bayesian Inference, Phys. Rev. Lett. **122**, 225701 (2019).

[14] A. P. Bartók, R. Kondor, and G. Csányi, On Representing Chemical Environments, Phys. Rev. B **87**, 184115 (2013).

[15] M. Parrinello and A. Rahman, Crystal Structure and Pair Potentials: A Molecular-Dynamics Study, Phys. Rev. Lett. **45**, 1196 (1980).

[16] M. Parrinello and A. Rahman, Polymorphic Transitions in Single Crystals: A New Molecular Dynamics Method, J. Appl. Phys. **52**, 7182 (1981).

[17] M. Pols, G. Brocks, S. Calero, and S. Tao, Temperature-Dependent Chirality in Halide Perovskites, J. Phys. Chem. Lett. **15**, 8057 (2024).

[18] A. Togo, First-principles Phonon Calculations with Phonopy and Phono3py, J. Phys. Soc. Jpn. **92**, 012001 (2023).

[19] A. Togo, L. Chaput, T. Tadano, and I. Tanaka, Implementation Strategies in Phonopy and Phono3py, J. Phys. Condens. Matter **35**, 353001 (2023).

[20] M. Hamada, E. Minamitani, M. Hirayama, and S. Murakami, Phonon Angular Momentum Induced by the Temperature Gradient, Phys. Rev. Lett. **121**, 175301 (2018).

[21] H. Chen, W. Wu, J. Zhu, Z. Yang, W. Gong, W. Gao, S. A. Yang, and L. Zhang, Chiral Phonon Diode Effect in Chiral Crystals, Nano Lett. **22**, 1688 (2022).

[22] L. Zhang and Q. Niu, Angular Momentum of Phonons and the Einstein–de Haas Effect, Phys. Rev. Lett. **112**, 085503 (2014).

[23] Y.-J. Choi and S.-H. Jhi, Divergence of Phonon Angular Momentum Driven by Temperature and Strain, Phys. Rev. B **106**, 094311 (2022).
